# Towards Point-of-Care Diagnostic and Staging Tools for Human African Trypanosomiaisis

**DOI:** 10.1155/2012/340538

**Published:** 2012-03-27

**Authors:** Enock Matovu, Anne Juliet Kazibwe, Claire Mack Mugasa, Joseph Mathu Ndungu, Zablon Kithingi Njiru

**Affiliations:** ^1^School of Veterinary Medicine, Makerere University, P.O. Box 7062, Kampala, Uganda; ^2^Foundation for Innovative New Diagnostics, Budé 16, Avenue 1202, Geneva, Switzerland; ^3^School of Veterinary Sciences, The University of Queensland, Gatton Campus, Brisbane, QLD 4343, Australia

## Abstract

Human African trypanosomiasis is a debilitating disease prevalent in rural sub-Saharan Africa. Control of this disease almost exclusively relies on chemotherapy that should be driven by accurate diagnosis, given the unacceptable toxicity of the few available drugs. Unfortunately, the available diagnostics are characterised by low sensitivities due to the inherent low parasitaemia in natural infections. Demonstration of the trypanosomes in body fluids, which is a prerequisite before treatment, often follows complex algorithms. In this paper, we review the available diagnostics and explore recent advances towards development of novel point-of-care diagnostic tests.

## 1. Introduction

Human African trypanosomiasis (HAT), also known as sleeping sickness, is a parasitic disease caused by flagellated protozoa of the genus *Trypanosoma*. Transmitted by the Tsetse fly (*Glossina* sp), HAT is endemic in rural sub-Saharan Africa that offers suitable habitat for the vectors, mainly riverine forests and savanna. The disease occurs in two forms, namely, the chronic type attributed to *T. brucei gambiense* (Gambian sleeping sickness) that is prevalent in central and west Africa as well as the acute (Rhodesian) form due to *T. b. rhodesiense* in east and southern Africa. HAT has been a major public health problem since colonial times when it wiped out entire villages in hard to reach areas of Africa. This situation was reversed through vigorous mass screening campaigns and vector control. However, during the wave of political instability and civil strife that swept sub-Saharan Africa in the later part of the last century, there was an upsurge in HAT incidence. Out of the known 36 endemic countries, over 90% of the cases were reported from Angola, Democratic Republic of Congo, southern Sudan and Uganda. Presently, HAT incidence has registered a steady decline over the past decade. Previous estimates indicated an annual incidence of about 70,000 cases [1, 2]; in 2006, the DRC had the highest incidence reported as 8,023 followed by Angola (1,105) out of the overall 11,382 for that year [3]. By 2009, the numbers had dwindled even further [4]. Although there may be underreporting, this trend indicates that elimination of HAT is within reach, at least in a few countries that continue to report no cases.

The disease is invariably fatal if left untreated and progresses through 2 stages: the early hemolymphatic stage, also known as stage I and the late meningoencephalitic stage (stage II) when the trypanosomes penetrate beyond the blood-brain-barrier. In addition, HAT exhibits 2 distinct forms associated with the causative subspecies: *Trypanosoma brucei gambiense* infection manifests as a chronic condition that claims its victims after several months, or even years. On the other hand, *T. b. rhodesiense* causes an acute infection that may kill patients within a few weeks [5]. HAT is among the so-called neglected tropical diseases (NTDs) that afflict rural poor communities in the developing world. In terms of disability-adjusted life year (DALY) estimations, this disease ranks high among other NTDs such as ascariasis, schistosomiasis, leishmaniasis and hookworm infection [6, 7]. In another study carried out in an endemic area of eastern Uganda, the burden attributed to *T. b. rhodesiense* HAT was reported to be only second to malaria, despite the former's relatively low incidence [8]. Mandatory and long durations of hospitalization render HAT an important problem within the usually resource poor health facilities, adding to logistical requirements of the already constrained services.

Control of HAT largely relies on chemotherapy for which there are only a few drugs, all associated with unacceptable toxicity and complex treatment regimens. The drugs are particularly old, the latest regimen being NECT that combines nifurtimox that is normally indicated for Chagas' disease (*T. cruzi*) and eflornithine, which was initially developed as an anticancer agent. Presently, only one drug Fexinidazole is in phase I clinical trials [9], while another compound (an oxaborole) has recently passed proof of principle in experimental infections [10]. The high toxicity of trypanocidal drugs dictates that trypanosomes must be demonstrated and the disease stage determined before any prescriptions can be made (late stage disease requires drugs that can cross the blood-brain-barrier). Indeed HAT case definition is by demonstration of trypanosomes by microscopy in fresh or stained body tissues, namely, blood, lymph, or cerebrospinal fluid (CSF). The available methods for this are tedious and suffer low sensitivity [11]. Availability of serological screening methods is, therefore, important to identify individuals on whom to execute the laborious parasitological methods. For this, the Card Agglutination Test for Trypanosomiasis (CATT) [12] has been a success story in diagnosis of *T. b. gambiense* HAT. However, most *T. b. rhodesiense* do not express the antigen on which the CATT is based. To date there is no field adapted serological test for this form, a major setback to screening in *T. b. rhodesiense* endemic areas.

The field of diagnostics for HAT has largely lagged behind; the tendency has been more of modifications of preexisting techniques than development of entirely new ones. Here we review the available options and explore recent advances towards development of novel point-of-care diagnostics.

## 2. Available Approaches to HAT Diagnosis

As noted above, HAT case identification relies on demonstration of trypanosomes in body fluids. The major obstacle to this is the characteristically low parasitaemia, especially in *T. b. gambiense *HAT, necessitating concentration methods that add to the complexity of the diagnosis. Serological screening, therefore, plays a pivotal role in identifying individuals for parasitological manipulations. The CATT [12] is the only available test for this and is the entry point to all the different diagnostic algorithms that vary from country to country, or even between regions of the same country depending on availability of the different parasitological tests as well as personnel to execute them. The CATT is an antibody test based on the LiTat 1.3 gene that is present in *T. b. gambiense*, although some stocks from Cameroon could reportedly not be detected due to absence of this gene [13]. Another report indicated that some stocks may be missed when they do not express the gene that is otherwise present [14]. The classical CATT kit consists of lyophilised antigen supplied in units of 50 tests, whose integrity relies on the cold chain. The latest advance in this area has been development of a thermostable version in units of 10 tests for wide coverage of and practicality [15]. The shortcoming of CATT, like any other antibody test, is that it does not necessarily indicate active infection. In addition, false positives are common, arising from cross reactivity with other pathogens including malaria or even transient nonhuman infective trypanosomes [12] that get inoculated into the bloodstream.

## 3. Parasitological Methods

Typically, all diagnostic algorithms start with CATT screening, followed by parasitological confirmation and staging [11, 16]. Available techniques have been extensively reviewed elsewhere [11]. The Haematocrit Centrifugation Technique (HCT) [17] is the test of choice for demonstration of parasites since it concentrates trypanosomes at the buffy coat area where they can be located by microscopy. The detection limit of this method is 500 trypanosomes/mL of blood [11]. This is no problem for *T. b. rhodesiense *that is in many cases easily detectable from wet films or stained thick smears, but some *T. b. gambiense* cases could still be missed due to low parasitaemia. Sensitivity of this method can be improved by examining several capillaries from the same patient. A more sensitive concentration method is the mini-Anion Exchange Centrifugation Technique (mAECT) that was adapted for field use over 3 decades ago [18]. This has subsequently been modified into formats that facilitate easy assembly, execution, and subsequent visualisation of the trypanosomes [19]. The kits are now produced and can be ordered from the Institut Nationale de Recherche Biomedicale (INRB), in the DRC. Latest additions to trypanosome concentration involve differential lysis of red blood cells to leave behind intact trypanosomes and white cells, followed by centrifugation. This greatly improves sensitivity; up to 3 mL blood can be concentrated to a pellet that is used to prepare smears that could be examined as wet or fixed and stained. For convenience, homemade or commercially available lysis solutions can be used to process the sample (see http://www.finddiagnostics.org/programs/hat/find_activities/parasite_detection/parasite_concentration).

## 4. Parasite Staining

**Figure 1:**
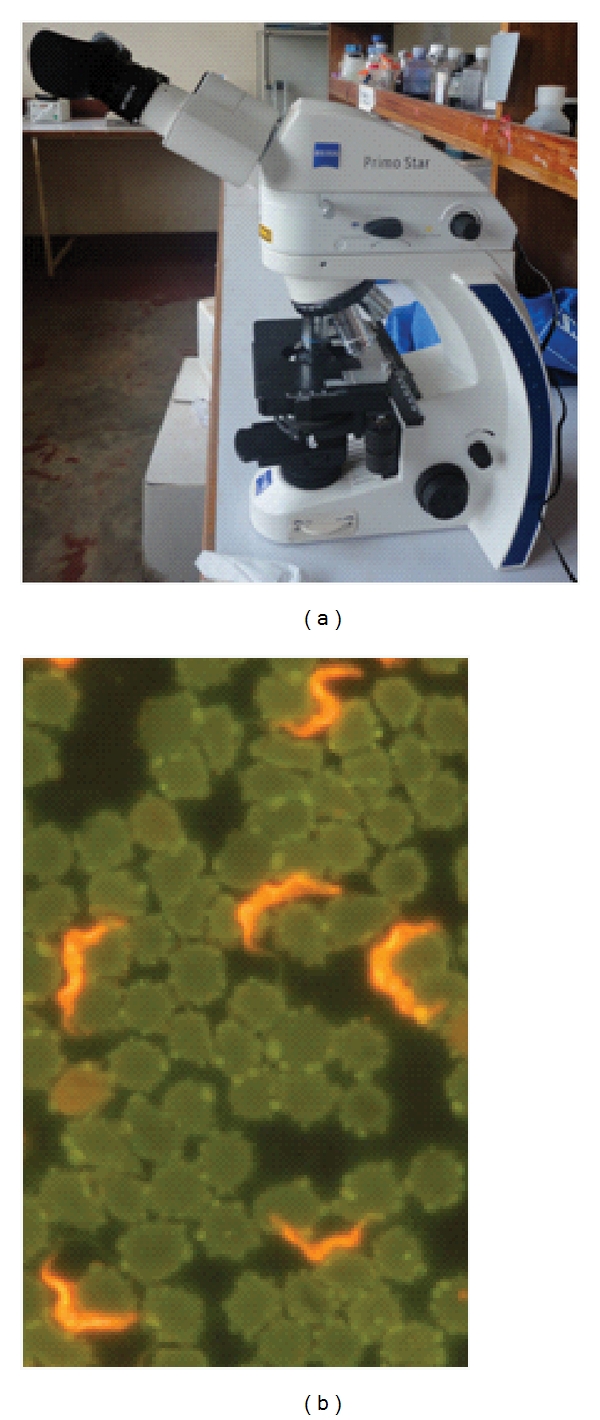
(a) the Primo Star iLED microscope has a UV light source that is beamed onto the slide from above, in addition to the classical white light from below, with a knob for interchange between UV and white light. (b) in presence of UV light, acridine orange stained trypanosomes fluoresce against a dark background, making it easy to detect the parasites.

The stain of choice has for a long time been Giemsa, few laboratories use field stains for trypanosome diagnosis. However during the 1990s, acridine orange was used as a good alternative since it enables trypanosomes to fluoresce in presence of UV light. Thus, the quantitative buffy coat technique was devised [20] and subsequently used albeit at a low scale in screening programs. The major issue here was the expensive microscope that was available then, coupled with the requirement for a dark room, features that made the technique nonfeasible at a typical treatment centre in endemic countries. The Foundation for Innovative New Diagnostics (FIND) has recently revisited this technique and, together with Carl Zeiss, developed a simple Primo Star iLED microscope (Figure 1), which offers options for both fluorescence and bright field microscopy. The ultrabright light-emitting diode (LED) technology exploited for this microscope provides a cheap, long-lasting light source (diode lifespan >10,000 hours). In addition, this microscope does not require a dark room and can be solar-powered since it consumes little energy. Besides, acridine orange typically stains all nucleated cells, so the iLED microscope can be applied to other haemoparasites that may coexist with HAT in the same endemic areas. The iLED microscope is now undergoing field evaluation in Uganda and the DRC.

## 5. Disease Staging

This is a critical step in diagnosis that guides the choice of treatment, given that drugs like pentamidin and suramin cannot cross into the central nervous system in sufficient amounts, and are, therefore, only prescribed for early (stage I) disease. HAT staging relies on the lumbar puncture to obtain CSF that is used to determine white blood cell counts, which rise over 5/mm^3^ at onset of the late stage disease. Live trypanosomes in the CSF are demonstrated after single [21] or double centrifugation of CSF [22], where the motile parasites are located in the cell pellets. This method too has been further developed into the modified single-centrifugation (MSC) technique [19] to ease parasite detection, and kits can be ordered from the INRB. Production of mAECT and MSC kits in the DRC are fruits of collaborative efforts between the Institute of Tropical Medicine (ITM) in Antwerp, FIND and INRB, a model for empowering endemic countries to produce some of the vital supplies for the national control programs.

## 6. Current and Upcoming Novel Molecular Diagnostics

DNA-based diagnostic tests have been pursued since the 1980s when species-specific probes were first described [23, 24]. Upon the advent of the polymerase chain reaction (PCR) for in vitro DNA amplification [25], species-specific oligonucleotide primers were designed for detection of trypanosomes [26, 27]. Since then, numerous targets within the trypanosome genome have been explored [28], including subspecies-specific PCRs that can detect *T. b. gambiense* or *T. b. rhodesiense* [29–31]. The major shortcoming with the latter is that their targets are single-copy genes [29–31], such that in several laboratories nested PCR is used [32–35] to maximise chances to obtain signals. Therefore in areas where the geographical distribution of the two forms of HAT is clearly known, PCR aimed at multicopy targets should be preferred for maximum sensitivity. As can be expected, PCR is a highly sensitive and specific method for identification of cases. However, its requirement for well-equipped laboratories coupled to a well-established cold chain to preserve reagents inevitably dictates that PCR will for a long time be based at reference laboratories far away from rural treatment centres. Nevertheless, PCR remains invaluable for epidemiological studies, including disease mapping and monitoring for drug resistance for compounds whose markers are known.

The latest approach to reduce equipment requirements for PCR was detection of products by oligochromatography (OC), which eliminates the need for electrophoresis that usually follows the thermocycling. For this, a PCR-OC test targeting the 18S rRNA gene was developed and displayed satisfactory sensitivity during the proof of principle [36]. PCR-OC still requires a thermocycler.

## 7. Nucleic Acid-Based Amplification (NASBA)

Another DNA amplification method that can be coupled to chromatography is the nucleic acid-based amplification (NASBA) of RNA [37]. This differs from conventional PCR in that it is run at isothermal conditions, therefore, replacing the thermocycler with much less expensive equipment (a heating block or water bath is sufficient). This is an advantage that would bring NASBA closer to the field situation. However, its major shortcoming is the need for elaborate RNA purification procedures; such facilities for purification and preservation of the characteristically fragile RNA remain major obstacles for its use in the field.

**Figure 2:**
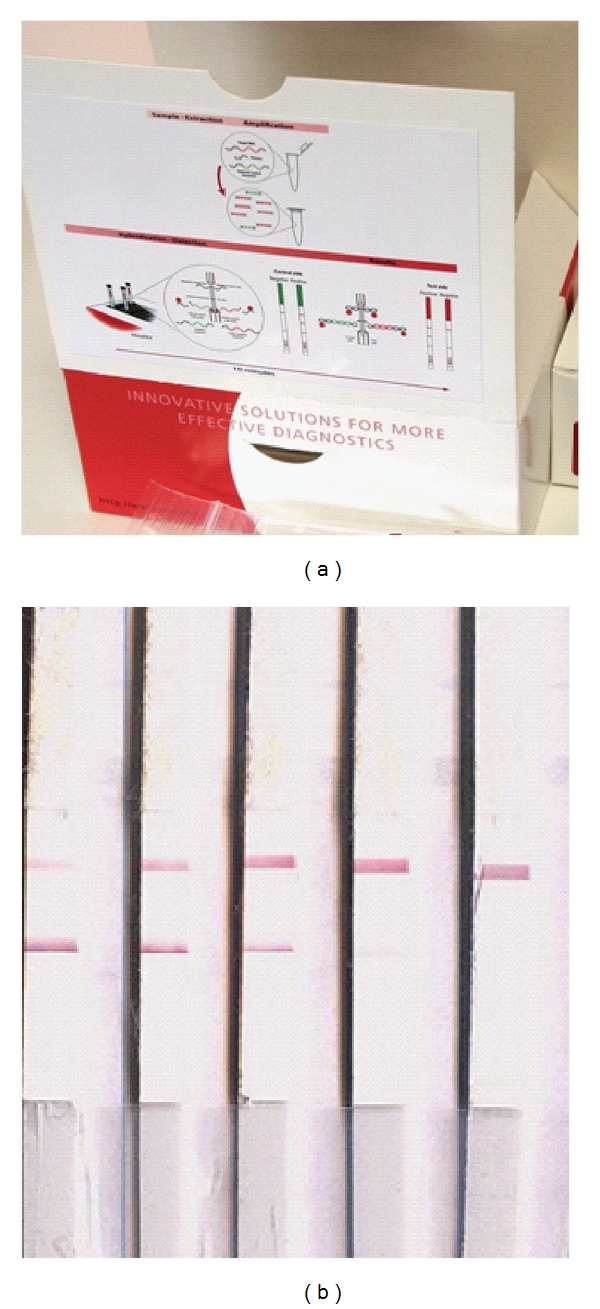
(a) sample Oligo-C test produced for diagnosis of HAT or Leishmaniasis by Coris Bioconcept. (b) results of NASBA-OC on *T. b. rhodesiense* cases from Eastern Uganda. Extreme left and right are the positive and negative controls, respectively. The 2 samples next to the positive control were clearly positive.

NASBA for trypanosome DNA amplification was first described in real time [38] and then extended to visualisation of the amplification product by OC [39]. The developed NASBA-OC was designed for the same target as PCR-OC but it amplifies the rRNA itself, thus is likely to be more sensitive since numerous RNA copies can be transcribed from a single gene. PCR and NASBA-OC were recently evaluated on clinical samples from endemic countries [40]. Indeed, the latter was more sensitive, as would be theoretically expected. The use of these techniques at least at reference laboratories will depend on how readily they are demanded by end users. Indeed, PCR-OC is available on demand (Figure 2), while a related test for Leishmaniasis (PCR-OC Leish) was commercialized over 2 years ago and is now marketed as Leish OligoC (http://www.corisbio.com/Products/Leishmania.php). Due to over cost, there are many challenges to commercialization of NASBA-OC, a typical example of obstacles to development of interventions for diseases that affect the poor. PCR and NASBA-OC tests for HAT and Leishmaniasis were developed in an EU funded collaborative project “TRYLEIDIAG” under the EUFP7 program.

## 8. The Loop-Mediated Isothermal Amplification (LAMP) Technique

**Figure 3:**
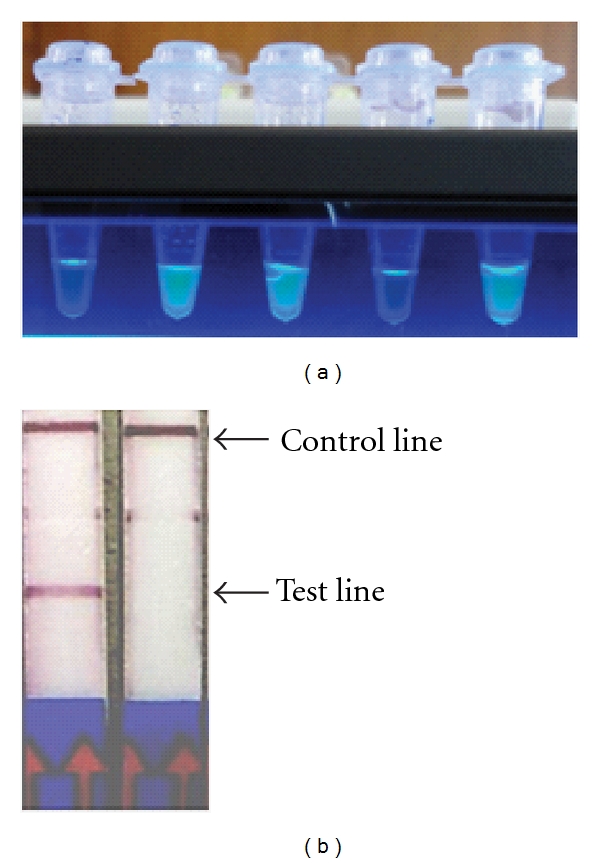
(a) visualisation LAMP products by fluorescence. To the extreme left and right are the negative and positive controls respectively. The 2 samples next to the negative control were from *T. b. rhodesiense* HAT cases, the one next to positive control is from a non-HAT infected individual. (b) detection of RIME LAMP amplification product using doubled labelled Milenia Genline Hybridetect 2 lateral flow strips. Product detection is achieved through hybridisation of fluoresceine isothiocyanate (FITC)-labeled probe with biotinylated LAMP product followed by combination with gold-labeled anti-FITC forming a triple-labeled complex which moves up the strip and is captured by an immobilized biotin-binding protein (test line). The nonhybridized FITC probe binds to the gold-labeled anti-FITC to form a double complex without biotin and is trapped at the control line. The faint line between the test control lines is nonspecific binding at DIG test line.

Among the latest developments in the area of molecular diagnostics is the loop-mediated isothermal amplification (LAMP) of DNA. It was first described by Notomi et al. [41] and has to date been pursued for diagnosis of several diseases. LAMP has been applied to detection of HAT in several studies, starting with that targeting the Para-Flagellar Rod protein A (*PFR A*), which is diagnostic for *T. brucei*. In that study analytical sensitivity of up to 100 times compared to the PCR was reported [42]. Subsequently, Thekisoe et al. [43] described LAMP targeting the 5.8S rRNA-internal-transcribed spacer 2 (ITS2) gene for detection of *T. b. gambiense*, reporting analytical sensitivity of up to 0.01 trypanosome in the tested sample. The following year, Njiru et al. [44] published LAMP that amplifies the random insertion mobile element (RIME) that is diagnostic of the subgenus *Trypanozoon*. The sensitivity observed was 0.001 trypanosomes/mL, thanks to the high copy number of RIME, reportedly 500 copies/haploid genome [45]. Subsequent work was targeted to the serum resistance-associated (SRA) gene that is specific for *T. b. rhodesiense*; the resultant LAMP test could detect 10 trypanosomes/mL of blood [46]. Subsequently, RIME- and SRA-LAMP were compared to SRA PCR for detection of DNA from *T. b. rhodesiense* patients spotted on Flinders Technology Associates (FTA) cards and stored for up to 2 years, where LAMP displayed superior sensitivity [34]. Most recently, LAMP for detection of *T. b. gambiense* has been described [47]. Despite the ultrasensitive nature of LAMP, the only shortcoming is that most assays rely on indirect methods for detection of amplification products. For this, several formats are available ranging from DNA intercalating dyes to measurement of turbidity. Comparative evaluation of some of them [48] indicates that their correct interpretation can vary, and the results could therefore be subjective. Thus, the ultra sensitivity of LAMP could be compromised by inappropriate visualisation of products. As a solution to this, Njiru [49] has recently devised a lateral flow test that specifically detects genuine amplification products (Figure 3).

Among the recently devised molecular tests, LAMP has highest prospects for application at or nearest to the treatment centre. It requires just a water bath or heating block that can be maintained at 60–65°C for 30–40 minutes, and the results can be readily visualised by naked eye. Besides, there is no need for the elaborate template purification methods that are a must for PCR and NASBA. Because of these advantages, the future of LAMP as a point-of-care test is presently being pursued by FIND. This test will be useful for screening and possibly also as a test of cure that could shorten the mandatory 18–24 month posttreatment follow-up period. A prototype LAMP kit for HAT was launched by Eiken Chemical Company in Japan in July 2011 and performance evaluation studies at treatment centers spearheaded by FIND are ongoing.

## 9. The Quest for HAT Biomarkers

Biomarkers for HAT have gained attention over the past decade. They stand a good chance of being exploited to develop tests that would not require the cold chain so they can have wide penetration to the treatment centres. The formats of choice would be dipsticks or lateral flow tests that would provide results within minutes of application of the sample. Such tests that involve limited or noninvasive means to obtain the test sample will be most successful. The ultimate aim should be to remove the requirement for the dreaded lumbar puncture that is presently a major obstacle to treatment seeking and followup. The search for HAT biomarkers has been explored from 3 fronts: biomarkers for disease severity, staging, and tests of cure. Biomarkers for disease severity have been pursued with the aim of identifying immunological responses that exacerbate infection or soluble products from the trypanosomes that directly contribute to pathology. The aim here would be to identify those in order to design molecules that could be used to offer supportive treatment with a view to facilitate patient recovery. Morty et al. [50], for example, demonstrated that administration of trypanosomal oligopeptidase inhibitors improved the survival rate of mice infected with *T. b. brucei*. Similarly, Mamani-Matsuda et al. [51] used quercetin and found that it induced dose-dependent decreases in the levels of TNF-*α*and nitric oxide and induced actual death of the parasites. Nitric oxide is among the products of activated human macrophages and contributes to central nervous system pathology in late stage HAT via its toxic derivatives or the ensuing oxidative stress.

## 10. Biomarkers for HAT Staging

Biomarkers for disease staging have been applied for decades despite presence of a limited number of known candidates. These are elevated CSF protein and white cell counts that have traditionally been used in addition to actual demonstration of the trypanosomes. Subsequently, the observed raised IgM in late-stage HAT was exploited to develop a Latex IgM test that can be used for staging [52]. Later work has explored inflammation-related proteins and markers of brain damage in the CSF with the belief that these should be elevated upon invasion of the brain compartment by the trypanosomes. Indeed Hainard et al. [53] demonstrated that a combination of CXCL10 (a 10kDA interferon-gamma-induced protein, also known as IP-10), CXCL8 (Interleukin-8) and H-FABP (heart fatty acid binding protein) identifies late-stage HAT with 97% and 100% sensitivity and specificity respectively. To this list of markers have been added osteopontin and *β*-2-microglobulin [54], matrix metalloprotease 9, and intercellular adhesion molecule 1 [55] as well as neopterin (Tiberti et al., man in prep). These markers are all arrived at after analysing CSF, meaning that the lumbar puncture still has to be performed if they can be used as markers for staging. The future direction of research should be investigating which of the identified markers are elevated in plasma of late stage patients as well, although little promise so far has been obtained from the few candidates investigated in that context. Other fluids that do not involve any invasive sampling such as saliva, tears and urine should be investigated. Hardly any attention has to date been given to these. But Lejon et al. [56] previously demonstrated that trypanosome antibodies could be detected from saliva of HAT patients, indicating that noninvasive diagnosis of HAT is a possibility.

## 11. Molecular Diagnostics as Tests of Cure

The major obstacle facing national HAT control programs is the long posttreatment followup to confirm cure. In principle, all treated patients must return every 6 months for a period of 18–24 months for parasitological diagnosis to be carried out and trace any persisting trypanosomes, particularly in the CSF. The requirement for a lumbar puncture at every followup visit is a disincentive for patients to return, while the few willing to return may be deterred by the long distances they have to cover to reach the treatment center. Thus, without active case follow-up that is in itself very expensive, big proportions of treated patients never return for medical consultation. With this obstacle it is difficult to accurately determine the success on any control program, given that chemotherapy is the mainstay in this endeavor. Thus, any advance that points to tests that eliminates the lumbar puncture or at least reduce their frequency may be a big boost to followup. This might be achieved by application of ultrasensitive techniques to detect nucleic acids, other antigens of parasite origin or even host factors. Deborggraeve et al. [57] recently evaluated the PCR as a possible tool for followup. They demonstrated a poor sensitivity to detect relapsing patients by testing blood, while 20% of individuals declared as successfully cured by conventional parasitology still had signals indicative of trypanosome DNA in their CSF. This may mean that PCR in such a case is just acting as a measure of the rate of parasite DNA clearance from circulation, that the latter continues to diminish from blood even when live trypanosomes still exist in the CSF. Detection of DNA in CSF of cured patients up to 2 years after treatment means that the relatively stable nature of DNA in this case acts as a disadvantage such that DNA targeting techniques might not be useful in followup. The remaining option in this case would be to investigate RNA targeting diagnostics such as NASBA-OC. Given its relatively less stable nature, it would be more likely that any observed signals emanate from RNA that has presently or just recently been transcribed from live trypanosomes. Could the observation of dwindling DNA signals in blood of relapsing patients imply that trypanosomes that colonize the CSF never relocate backwards into the bloodstream? For it is generally believed that all available treatment regimens possibly attain drug concentrations in blood that are way above the effective doses, and that the “resistant” trypanosomes are favored to survive in CSF where just a fraction of the drug, concentrations observed in blood are able to establish. Some evidence to support this “one way” traversal comes from the observation that in melarsoprol relapse patients, trypanosomes are almost exclusively observed only in CSF [58, 59]. We have also made a similar observation in patients relapsing after eflornithine (Matovu et al., manuscript in preparation). Alternatively it is possible that they relocate but fail to reestablish in blood, given that their traversal in CSF is facilitated by massive adaptations to this relatively poor medium to the extent that they may fail to cope when they return to the blood system. This is surprisingly not the case in the mouse model, where trypanosomes hiding in the brain were reported to recolonize the blood system after treatment [60].

## 12. Biomarkers as Tests of Cure

Another option to consider for tests of cure are biomarkers, if candidates that drastically and consistently diminish from circulation after successful treatment can be identified. Several attempts have been made in this direction. Lejon et al. [61, 62] demonstrated that CSF cells, IL-10, IgM, and protein concentration all remain elevated in relapsing patients, while they continue to decline in successfully treated individuals. In a similar study, Ngoyi et al. [63] proposed an algorithm for reducing the follow-up duration that also combines determination of CSF IgM and white cell counts. Obviously, it would be added advantage if such markers can be traced in blood or noninvasively from other body fluids.

## 13. Conclusions and Way Forward

There are some recent advances towards identification of candidates that could be used to develop point-of-care diagnostics and staging tools. However, this area of research has advanced at a rather slow pace despite the known fact that accurate diagnosis is key to the success of any HAT control program. Even at this point when we can imagine elimination of HAT as a possibility in some regions, highly sensitive and widely applicable diagnostic tools will be at the centre of any such attempt. High precision diagnostics are required to screen the entire populations in order to fish out the last cases or animal reservoirs. It is surprising that since the development of the CATT in the late 1970s, only one potential replacement has been developed. The lateral flow Rapid Diagnostic Test (RDT) that has just been developed is now undergoing evaluation in some *T. b. gambiense* endemic countries (see http://www.finddiagnostics.org/programs/hat/find_activities/serodiagnosis.html). The other report is an attempt improve the CATT by development of synthetic peptides [64] to use in the place of native antigens that tend to cross-react, giving the characteristic false positives associated with the test. Meanwhile, a serological screening test for *T. b. rhodesiense* is still missing.

Therefore, much as recent attempts to identify biomarkers and genomic sequences to exploit for new diagnostics should be appreciated, there is much more effort required if we are to realize continued HAT suppression. As mentioned elsewhere, the current needs are not only novel point of care diagnostics, but also those that can eliminate the need for a lumbar puncture should be given priority. It should be borne in mind that staging will be required for as long as there is no safe drug that is effective against both stages of the disease. Thus, patients who shun followup because of the lumbar puncture remain potential reservoirs from which trypanosomes that have been exposed to treatment can be transmitted, thereby spreading drug resistance.

The new fluorescence LED microscopy and the possibility to concentrate blood much more effectively than ever before are good developments in demonstration of parasites in cases with extremely low parasitaemia. Their evaluation in *T. b. gambiense* areas needs to be expedited to show if incidences of the so-called aparasitaemic cases [65] can be curbed. At the same time, hand-held prototypes built with similar technology as the LED microscope should be explored, as they would allow for wider coverage of the diagnostic services. Lastly, the need for an RDT type capable of detecting *T. b. rhodesiense *cannot be over emphasized. Actually, this on its own can be argued to be the only obstacle hindering elimination of the acute form of HAT.
